# Bacterial Vaginosis (BV) and Vaginal Microbiome Disorders in Women Suffering from Polycystic Ovary Syndrome (PCOS)

**DOI:** 10.3390/diagnostics14040404

**Published:** 2024-02-12

**Authors:** Izabela Chudzicka-Strugała, Iwona Gołębiewska, Beata Banaszewska, Mateusz Trzciński, Grzegorz Brudecki, Wael Elamin, Barbara Zwoździak

**Affiliations:** 1 Department of Medical Microbiology, Poznan University of Medical Sciences, Rokietnicka 10, 60-806 Poznan, Poland; ichudzicka@vp.pl (I.C.-S.); barbara.zwozdziak@gmail.com (B.Z.); 2 Poznan’s Academy of Medicine, Bulgarska 55, 60-321 Poznan, Poland; 3 Department of Laboratory Diagnostics, Poznan University of Medical Sciences, Szamarzewskiego 82/84, 60-569 Poznan, Poland; bebanaszewska@gmail.com; 4 Department of Infertility Diagnostics and Treatment, Poznan University of Medical Sciences, 61-701 Poznan, Poland; mtrzcinski13@gmail.com; 5 Group 42 (Healthcare), Masdar City, Abu Dhabi P.O. Box 112778, United Arab Emirates; grzegorz.brudecki@g42.ai (G.B.); wael.elamin@g42.ai (W.E.)

**Keywords:** bacterial vaginosis, PCOS, vaginal microbiome disturbances, vaginal dryness, vaginal biocenosis (microbiome), *Lactobacillus* elimination, *Gardnerella vaginalis*, clue cells

## Abstract

**Introduction:** Polycystic ovary syndrome (PCOS) is a multifactorial, heterogeneous endocrine and metabolic disorder in women. Due to its association with the menstrual cycle and fertility disorders, the importance of this problem is emphasized especially in patients of reproductive age. Based on a number of analyses, the effect of PCOS on altering the diversity of the microbiome (e.g., intestinal or vaginal) is suggested. Vaginal dysbiosis can result in BV (bacterial vaginosis). The purpose of this study was to assess the prevalence of BV in patients with PCOS, as well as to determine the most reliable diagnostic factors. **Material and Methods:** Retrospective analysis of microbiological findings (2018–2022) of PCOS patients (*n* = 594) of reproductive age. The present analysis focused on the results of patients with PCOS (*n* = 380) and vaginal discharge with pH ≥ 4.4 and suspected BV. Biological material was a vaginal swab/vaginal secretion. The most commonly used routine methods for assessing BV were the Amsel analysis and the Nugent scoring system. **Results:** Patients with PCOS and vaginal fluid pH ≥ 4.4 and suspected BV (*n* = 380) accounted for 64% of all PCOS patients (*n* = 594). The relationship between pH and detection of “clue cells” showed significant dependency and increased with leukocytes. The pH measurement also showed dependency on high *G. vaginalis* counts. In addition, the elimination of lactic acid bacteria (LAB) in vaginal secretions was associated with an increase in the number of leukocytes with increasing pH values. A marked increase in *G. vaginalis* was found in more than half (56.8%) of PCOS women (*n* = 380) with suspected BV. No dependency was observed between the absence of LAB and the diagnosis of BV on a positive *G. vaginalis* culture. Of the *n* = 380 patients with PCOS, 191 (50%) had a Nugent score ≥ 7 positive for BV. No dependency was observed between the number of patients with *Candida* sp. in vaginal secretions and pH, BV (with clue cells), or elevated leukocyte levels. The LRM was adjusted and the statistical model represented by the following formula was obtained: log(*p*/(1 − *p*)) = −1.18 + 1.24 × Group4.6 + 1.08 × Group4.8 + 1.66 × Group5.4. **Conclusions**: Based on the present analysis, BV appears to be more common in patients with PCOS than in the non-PCOS population. Chronic inflammation in PCOS patients and abnormalities in the vaginal microbiome may predispose to the development of BV. In women with PCOS, BV may be one of the unrecognized causes of infertility or complications of pregnancy. Despite the potential link between PCOS and the development of BV, the extent to which this syndrome contributes to vaginal dysbiosis and reproductive complications requires further study.

## 1. Introduction

One of the most frequently diagnosed (5–20%) heterogeneous endocrine and metabolic disorders of women in reproductive age is polycystic ovary syndrome (PCOS) [1]. Due to its relationship with disturbances in menstrual cycles and fertility, especially in women of reproductive age, it has attracted the attention of researchers [2]. Among the main endocrine disorders are excessive ovarian androgen secretion or activity, lack of ovulation and infertility, and abnormal insulin activity. PCOS is also associated with several complications including menstrual disorders, infertility, hirsutism, acne, obesity, or metabolic syndrome [3], and affects many aspects of women’s health with long-term effects far beyond reproductive age [4].

In recent years, the crucial importance of the microbiome and its balance as an integral component of the human body in regulating the homeostasis of many systems and maintaining health has been emphasized. Based on its location, the microbiome of the vagina, intestines, oral cavity, respiratory system, and skin has been distinguished. In the case of PCOS patients, many studies indicate changes in the diversity of the microbiome (including the gut and vagina), resulting in an imbalance or even dysbiosis compared to healthy women [2]. It is also suggested that an imbalance of the microbiome is involved in the genesis of PCOS, leading to the development of low-grade chronic inflammation. In addition, a reduction in diversity and excess of certain bacterial species leads to metabolic disorders [4,5]. The interactions of the vaginal microbiome with clinical symptoms of PCOS are not fully understood. However, some studies in PCOS patients indicate a reduced contribution of *Lactobacillus* spp. (especially *L. crispatus*) to the vaginal microbiome and a higher prevalence of microorganisms such as Prevotella, for example, and atypical pathogens (e.g., Mycoplasma, Chlamydia) [6]. Moreover, the environment of the vagina and cervical canal of patients with PCOS, and reduced fertility, in addition to reduced numbers of *Lactobacillus* spp., showed an increase in favorable conditions promoting the growth of *Gardnerella* sp. and other pathogenic microorganisms, which may result in the development of bacterial vaginosis (BV) [7]. BV remains an important gynecological and obstetric problem. Although BV pathophysiology and symptoms are relatively well-studied, it is not always recognized by clinicians. Despite more than 60 years of knowledge about the problem of BV, scientists are still searching for its clear causes and complications [8]. The development of BV is believed to be associated with states of vaginal dysbiosis, as a result of the elimination of lactic-acid-producing bacteria, such as *Lactobacillus* spp., and the significant proliferation of *Gardnerella vaginalis* and other highly diverse anaerobic bacteria, such as *Mobiluncus* spp. and *Bacteroides* spp. [9]. Since BV can also be asymptomatic, with only an overgrowth of the vaginal microbiome without the presence of inflammation, choosing the best commonly available diagnostic method remains difficult [10,11].

An imbalanced vaginal microbiome seems to predispose women with PCOS to develop BV due to its commonness and recurrence [10]. It is worth paying special attention to this group of patients. Therefore, in the present study, we tried to highlight the occurrence of the BV problem based on a retrospective analysis (2018–2022) of the microbiological analysis results of patients with diagnosed PCOS syndrome.

## 2. Materials and Methods

In the diagnosis of BV, the importance of microbiological analysis of vaginal discharge, and the use of targeted rather than empirical therapy, are emphasized. The nature of BV may vary, and treatment based solely on clinical symptoms may not be accurate. Moreover, ineffective therapies may additionally disturb the vaginal microbiome and even increase the resistance of microorganisms to treatment [12]. Both clinical criteria and laboratory tests are used to assess the presence of BV [13].

### 2.1. Patients

The study was based on a retrospective analysis (2018–2022) of a group of 594 women diagnosed with PCOS (aged 23–39) based on the Rotterdam Consensus criteria for PCOS and who presented with at least two of the following symptoms: (1) oligo- or amenorrhea; (2) clinical or chemical hyperandrogenism; and/or (3) polycystic ovaries as determined by transvaginal ultrasonography [14]. Patients presented to a gynecologist for routine follow-up or because of uncomfortable clinical symptoms suggestive of potential BV. Thyroid disease, Cushing’s disease, diabetes mellitus, hyperprolactinemia, and congenital adrenal hyperplasia were exclusion criteria in the analyzed patients. Symptomatic patients reported for, e.g., redness, itching of the vulva, “abnormal”/watery/grey appearance of vaginal discharge, unpleasant/“fishy smell” of the discharge, vaginal burning and/or dryness, and swelling (Table 1). All the patients were sent for microbiological tests to assess the vaginal microbiome and targeted therapy was implemented.

### 2.2. Material and Methods for BV Diagnostics

The most used methods for assessing BV were the analysis based on Amsel’s criteria and the Nugent scoring system (Table 2).

In the case of the Amsel criteria, a BV is found when three of the four criteria are considered positive, as opposed to the Nugent criteria, which are based on the microscopic assessment of the presence of Gram-positive bacteria (*Lactobacillus* spp.) and Gram variables (associated with BV) with an assigned score. In the case of the Nugent method with the assessment of morphotypes in a smear of vaginal discharge, it is possible to detect the presence of BV even in the asymptomatic form [8,9,23,24,25,26].

**Figure 1:**
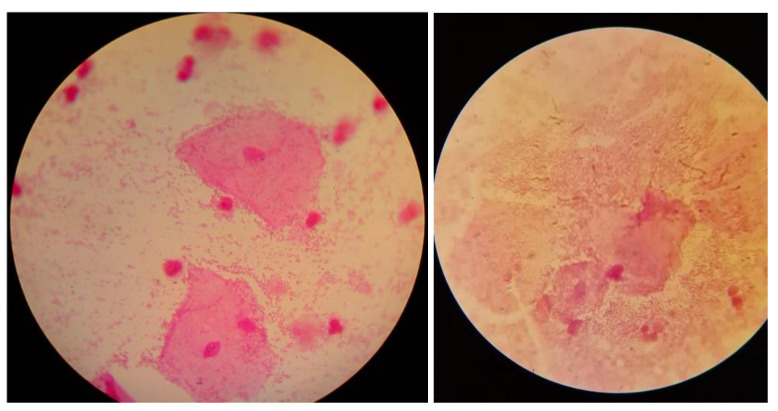
Examples of clue cells under a microscope (photos from authors’ resources).

The diagnostic material consisted of vaginal secretions and/or swabs from the posterior vaginal vault and lateral vaginal walls, taken on 5 separate sterile swabs. A preliminary macroscopic evaluation of the vaginal secretions was then performed based on Amsel’s criteria. The secretions were analyzed for the detection of a “fishy odor” using 10% potassium hydroxide (KOH)—the “whiff test”. A wet mount was directly prepared from the vaginal swab to detect “clue cells” and Trichomonas vaginalis, among others [27]. Moreover, a vaginal swab was directly smeared on a microscope slide for Gram staining preparation, and potential detection of “clue cells” (Figure 1) and further evaluation of bacterial occurrence and morphotypes using the Nugent score [13,28]. It is worth noting that in the absence of the classic BV presence of vaginal discharge, the presence of abnormalities in the vaginal microbiome is not ruled out, so further diagnostics should be pursued [13]. The pH of the vaginal discharge was measured using strips for gynecological pH examinations (3.8–6.5), either by directly touching the vaginal wall or after swabbing and applying the material to the pH strip [13,29]. The diagnostic material was also cultured for *Lactobacillus* spp. (Rogosa agar), *Gardnerella vaginalis* (*Gardnerella vaginalis* selective medium), *Candida* spp. (Sabouraud medium), and Trichomonas vaginalis (Trichomedium).

Finally, this analysis focused on a group of PCOS patients (*n* = 380) whose vaginal fluid pH showed an increasing trend, and in whom changes in the vaginal microbiome were observed and BV was suspected.

From a group of 594 results of patients diagnosed with PCOS, 380 were finally selected for analysis based on the criteria presented in Table 3.

## 3. Results of BV in Women with PCOS

A total of 594 gynecological patients with PCOS were selected for the presented retrospective research. However, this study focused on the analysis of patients (*n* = 380) with vaginal fluid pH ≥ 4.4 and suspicion of BV, who accounted for as much as 64% of the analyzed group of women with PCOS. In the remaining PCOS patients (*n* = 214), the pH of the vaginal secretion oscillated between 3.8 and 4.2. These women showed no BV “markers” and were negative for BV, with a Nugent score of 0–3, did not report any additional symptoms, and came for a gynecological examination for a routine check-up, e.g., before a planned pregnancy.

In the presented study, the relationship between pH and detection of “clue cells” resulted in significant dependency, which increased with leukocytes detected at a high count (>10) in the samples. Thus, the fraction of patients with clue cells detected at a high count was higher in the groups with higher pH. The elevated vaginal leukocyte count was found to be dependent on the increasing pH.

In accordance with accepted systems of routine BV assessment, the analysis of clue cells was considered helpful in diagnosing bacterial vaginosis (BV) in all patients tested, as there was a positive dependency found between the detected clue cells and high count of *Gardnerella vaginalis* [30,31]. The presence of clue cells compared with the high abundance of *Gardnerella* in different pH categories was also tested with the chi-squared test (Table 4). Here, χ^2^ (calculated) < χ^2^ (tabulated) or 5.88 < 7.84. Hence, we accept the null hypothesis. At the 5% level of significance, there is insufficient evidence to conclude that the population follows a distribution different from that predicted and is independent of random variables.

The logistic regression model (LRM) was also developed by transformation of the data into Boolean variables (presence and absence of clue cells) for each pH group (*n* = 4). The data matrix for the execution of the LRM was created. Each line corresponds to a sample (a total of 380 lines) and includes information on the pH group to which it belongs and whether it presents clue cells (1 = presence and 0 = absence) (Table 5).

The LRM was adjusted and the statistical model represented by the following formula was obtained:

log(p/(1 − p)) = −1.18 + 1.24 × Group4.6 + 1.08 × Group4.8 + 1.66 × Group5.4

The coefficients for the different pH groups represent the differences in the log odds and show how the one-unit increase in each group can change the log of odds.

Our results confirm the significant diagnostic value of clue cells detection. pH measurement shows a lower dependency on high *G. vaginalis* counts. The obvious imbalance of the bacterial microflora of the examined patients was confirmed by the increasing percentage of patients, with the elimination of lactic acid bacteria in the vaginal secretion associated with an increased leukocyte count with increasing pH value. The absence of lactic acid bacteria with increased levels of leukocytes in different pH categories was also tested with the chi-squared test (Table 6). Here, χ^2^ (calculated) < χ^2^ (tabulated) or 3.32 < 7.81. Hence, we accept the null hypothesis. At the 5% level of significance, there is insufficient evidence to conclude that the population follows a distribution different from that predicted and is independent of random variables.

An increase in *Gardnerella vaginalis* was found in more than half (56.8%) of women with PCOS in the analyzed group of patients (*n* = 380) with suspected BV. Interestingly, a low correlation (R^2^ = 0.50) was observed between the absence of LAB and the diagnosis of BV with positive *G. vaginalis* culture. Of the 380 PCOS patients, 191 (50%) had a Nugent score ≥7 positive for BV. On the other hand, the remaining patients fit into the Nugent scale (4–6) and were assessed as intermediate, although they reported ailments. This fact is consistent with the observations of other researchers regarding a number of potential factors affecting the imbalance of the vaginal microbiome and the complexity of the BV development process, as well as a not entirely unambiguous diagnosis based on standard diagnostic methods [32].

In addition, no correlation was observed between the number of patients with *Candida* sp. present in their vaginal fluid and pH, BV (with clue cells), or elevated leukocytes (Table 7). Based on the analyzed groups of PCOS patients, *Candida* species present in the vaginal microbiome did not automatically eliminate the growth of LAB, and in some patients both types were detected. The data presented in Table 5 showing the number of patients with clue cells, a high level of leukocytes, and absence of lactic acid bacteria were also tested with the chi-squared test. Here, χ2 (calculated) > χ2 (tabulated) or 13.54 > 9.48. Hence, we reject the null hypothesis. The *p*-value of 0.035 implies a 3.5% probability that the results could have happened by chance. The smaller the *p*-value, the more important (significant) the results are. A significant result is one where the null hypothesis is rejected. Therefore, we can conclude that at the 5% level of significance, the clue cells, Leu > 10, and LAB none are dependent.

## 4. Discussion

Bacterial vaginosis (BV) in women is a widespread, complex, and fairly well-known problem worldwide, although some areas remain unexplained. To date, a single direct etiological agent has not been defined, and BV is not classified as a sexually transmitted disease. However, the potential impact of BV on increasing the risk of infection and predisposition to the development of sexually transmitted diseases is highlighted [33,34]. As such, BV is considered an important aspect of public health due to the increased risk of sexually contracting transmitted diseases, including HIV, HSV, Chlamydia trachomatis, or Neisseria gonorrhoeae, among others [33,35,36,37].

### 4.1. Incidence of BV

BV is considered one of the most common causes of vaginal dysbiosis in women of reproductive age and the occurrence of abnormal vaginal discharge, often with an unpleasant “fishy odor”, although the course of the disease can be different and sometimes even asymptomatic. The incidence of BV is estimated to range from 5–15% to as high as 30–40% in the population of women of reproductive age [38,39,40,41,42]. In a study by Salah et al. (2013), the percentage of women with infertility and BV was significantly higher compared to that in fertile women (45.5% vs. 15.4%). In patients with PCOS, the prevalence of BV was as high as 60%, and in women with unknown causes of infertility, BV was estimated at more than 35% [43].

The present study focused on the analysis of a selected group of patients (*n* = 380) with PCOS and vaginal fluid pH ≥ 4.4. Hence, BV was suspected in 64% of the analyzed group of women with PCOS. However, an increase in *Gardnerella vaginalis*, potentially associated with the development of BV, was found in more than half (56.8%) of the PCOS women in the analyzed group of patients (*n* = 380) with suspected BV. Interestingly, in this group, 191 (50%) patients were positive on the Nugent scale ≥ 7, and the remaining patients were rated as intermediate (Nugent scale 4–6), although they reported symptoms suggesting BV. This fact confirms earlier considerations about the varied course of BV and the not always clear-cut findings and potential under-detection of the problem. Moreover, the results of our analysis suggest a higher rate of BV in patients with PCOS than is estimated in the population of healthy women.

### 4.2. Predisposing Conditions for BV Development

Predisposing factors for the development of BV remain relatively unclear, but some of them include the course of menstrual cycles, sexual activity, or even personal hygiene [26]. Increasing attention is therefore being paid to the importance of the composition of the microbiome in the vaginal environment and its interactions in preventing colonization by pathogenic microorganisms [2]. The importance of the dominance of *Lactobacillus* spp. (>70%) in the vaginal microbiome is emphasized due to their production of compounds with antimicrobial activity, e.g., hydrogen peroxide or bacteriocins, for benefits related to improving sexual and reproductive health and preventing the growth of pathogenic microorganisms [19,26]. Studies of the microbiome of the vagina, but also of the gut and oral cavity, suggest that, compared to healthy women, PCOS patients are more likely to experience inflammation, leading to destabilization of such a microenvironment [19,44,45]. Patients with PCOS have chronic low-grade inflammation. According to a new microbiological hypothesis, a clear correlation of pathological changes, dysbiosis and hyperandrogenemia, or insulin resistance, and the role of gut–brain axis mediators in the gut microbiome are emphasized. In the case of women with PCOS, the impacts of factors faced by patients, such as high triglyceride, fasting glucose, and insulin levels, metabolic disorders, or diabetes, on the composition and changes in the microbiome and the role of *Lactobacillus* spp. in infection control and metabolic balancing processes, are highlighted [44,45,46,47].

For women with PCOS, additional inflammatory factors may therefore exacerbate the complications associated with this syndrome. Therefore, in the case of PCOS patients, the importance of achieving a balanced microbiome, facilitating control of the symptoms, is emphasized. This is confirmed by studies conducted on animal models, but also based on the results of analyses using probiotic/synbiotic therapies in women with PCOS. Beneficial effects of *Lactobacillus* spp. have been demonstrated, e.g., on the regulation of intestinal microflora related to sex hormones [48,49]. A person’s microbiome plays an important role and is considered their “second genome” [2]. The vaginal microbiome is a dynamic system that is influenced by cyclic hormonal changes (mainly estrogen and the menstrual cycle), glycogen levels, changes in the vaginal epithelium, or sexual activity, among other factors. To date, many hypotheses have been developed regarding the dynamics of changes in the vaginal microbiome community, but they do not provide a clear answer. Hence, especially in the case of patients with PCOS, it is worth paying attention to the impact of this condition on the interactions taking place within the vaginal microbiome and its imbalance [26,50], especially since currently the data on the vaginal microbiome for patients with PCOS are quite limited.

### 4.3. Impact of Hormones

In a woman’s lifetime, the vaginal microbiome is modulated by hormonal changes, from prepubertal to postmenopausal, as well as bimonthly hormonal fluctuations. In addition, age, immune system status, and even ethnicity, lifestyle, and diet, and the use of medications such as antibiotics, as well as probiotic/synbiotic supplementation, also have an impact [51]. Sex hormones affect the mechanisms of antigen presentation, cytokine production, and immunoglobulin production and transport, but also the mucosa of the reproductive system, its immunity, and the stability and balance of the vaginal microbiome [6]. In the case of PCOS, endocrine disorders, hormonal changes, and, of course, the consequences of these processes, may play an important role in shaping the vaginal microbiome. Many studies in PCOS patients emphasize the presence of chronic low-grade inflammation pointing to important inflammatory factors, including those such as IL-18, associated with insulin resistance, obesity, or metabolic syndrome, and even predicting long-term cardiovascular mortality. In addition, it is important to pay attention to the effects of systemic inflammation when the vaginal microbiome is disrupted. Studies show that in such cases there is an increase in IL-8 and tumor necrosis factor alpha, which affect the hypothalamic–pituitary–ovarian axis through the blood and lymph [6,52,53,54]. An important element, therefore, is the prevention of BV and its complications, which is not easy due to the not-fully-explained interactions of the reproductive tract mucosa with the vaginal microbiome, as well as the multifactorial and dynamic changes in this process, both from a microbiological and immunological point of view [55]. In women with PCOS, hormonal disorders (including estrogen and progesterone) or irregular menstrual cycles, affecting periodic changes in the epithelium of the reproductive tract, play a particular role in the maintenance of the vaginal microecosystem [2]. Moreover, androgens have been shown to modulate the gut microbiome, which is associated with intestinal dysbiosis in women with PCOS and hyperandrogenism [49,56,57]. Women with high testosterone levels were found to have higher levels of gut microbiome diversity, similar to PCOS, and its additional impact on the vaginal microbiome. Moreover, a study by Hong et al. (2021) found that patients with PCOS, regardless of testosterone levels, had a higher prevalence of Gardnerella, the microbiome most associated with BV development, compared to healthy women without PCOS [6]. This may also suggest the possibility of a higher prevalence of BV in a group of women with PCOS. As an example, a study by Hong et al. (2021) in women with PCOS found an effect of testosterone levels on *L. crispatus* and *L. iners* populations, and a study by Lu et al. (2021) found a correlation between the number of *Lactobacillus* spp. and FSH levels. A high percentage of these microorganisms is maintained at normal levels, and when it decreases, an increase in the percentage of opportunistic microorganisms in the vaginal microbiome is found [6,44]. A decrease in the proportion of *Lactobacillus* spp., especially *L. crispatus*, in the vaginal microbiome of women with PCOS is accompanied by a significant increase in pathogenic microorganisms such as *Gardnerella vaginalis* and *Prevotella* sp., but also Mycoplasma and Chlamydia trachomatis. An example is the study by Gu et al. (2022) in PCOS patients of the effect of the length of menstrual cycles on the reduction in the percentage of *Lactobacillus* sp., while inoculating with pathogenic microorganisms [2,6,7,58].

PCOS syndrome causes the imbalance of the vaginal microbiome due to the elimination of *Lactobacillus* spp. and facilitates the growth of anaerobic and pathogenic microorganisms. Hence, PCOS syndrome can predispose, for example, to BV and resulting reproductive failure, preventing embryo implantation or fetal growth [7].

### 4.4. Impact of Immune Response

In the present study, the imbalance of the bacterial microflora of the analyzed patients was confirmed by the increasing percentage of patients with the elimination of lactic acid bacteria in the vaginal secretions with an increase in pH, in which an increase in the number of clue cells was also found. In the PCOS patients analyzed, there was a correlation with an increased number of leukocytes in the vagina along with an increase in pH. It is also worth noting that in addition to the involvement of microorganisms in the development of BV, the importance of the immune response also plays an important role [59]. In the development of BV and homeostasis of the vaginal mucosa, the role of cervical and vaginal immunity is indicated, which is influenced by many diverse factors, such as genetics, reproductive hormones, psychosomatic stress, diet, and even physical activity [59]. Thus, in the case of a condition such as PCOS syndrome, this aspect assumes particular importance.

It is suggested that in women with BV, the number of neutrophils in the vagina usually does not differ from that in healthy women. The immune response shows a significant increase in the vaginal interleukin IL-1beta, as well as a suppression of the pro-inflammatory response by hydrolytic enzymes produced by microorganisms, resulting in an impaired increase in IL-8 and precisely the absence of neutrophils in most BV patients [60], which has not been confirmed by our analysis. In the case of the PCOS patient group in our analysis, the increase in neutrophils could therefore be explained by the potential development of infections caused by non-culturable microorganisms that were not identified during the evaluation of the vaginal microbiome, which were predisposed by the state of developed or developing BV, and would require further investigation in patients. Interleukin testing is not included in routine analyses of the vaginal microbiome and possible identification of BV; hence, it was not performed in the presented PCOS patients. Currently, little research has emerged on the vaginal microbiome in patients with PCOS. However, it is worth paying special attention to the disorders associated with this syndrome both in terms of immunity, physiological homeostatic processes, and pathological dysbiosis, including the vaginal microbiome, which is potentially more likely to result in the development of BV.

Despite the prevalence and knowledge of BV pathophysiology, due to the etiology and pathogenesis of this process, some areas are unclear. Therefore, the need to develop metabolic biomarkers that are specific and unique to BV is emphasized. Studies by Aldunate et al. (2015) point to changes in metabolic pathways, redox homeostasis, and inflammatory pathways in the vaginal ecosystem due to the production of short-chain fatty acids (SCFAs) by bacteria associated with the development of bacterial vaginitis (BVAB), leading to vaginal dysbiosis. It is therefore crucial to comprehensively understand and clarify the mode of action of BVAB (bacteria associated with bacterial vaginosis) and host factors that lead to disruption of the vaginal microbiome and loss of lactic acid-producing microorganisms [26]. The introduction of more precise and detailed diagnostic tests, available on a wider scale, would enable faster, unambiguous diagnosis, especially in the case of early development of BV, which is particularly important in patients with PCOS.

### 4.5. Impact of Diagnostics Methods for the BV Recognition

In the analysis we presented, in some patients with PCOS, despite their reported alarming symptoms, the diagnosis of BV was not confirmed on the basis of microbiological examination as the relevant accepted criteria were not met. It can be suspected that the process was at such an early stage that it could not be detected by routine methods commonly used in assessing the vaginal microbiome. It may be worth expanding the diagnostic criteria for women with PCOS, given the potentially higher likelihood of developing BV and dysbiosis of the vaginal microbiome. In addition, the introduction of molecular testing into the routine diagnosis of the BV microbiome especially in PCOS patients could yield more conclusive results and provide a different perspective on vaginal dysbiosis and the potential development of BV, also in the context of diagnosing infertility in PCOS patients.

To date, much attention has been paid to the diversity of the gut microbiome in women with PCOS. It is worth noting the diversity and disruption of the vaginal microbiome and the potential implications these may have for patients with PCOS, both from the point of view of the impact of this syndrome on changes in the diversity of microbial composition, and the extent to which a microbiome with this localization affects other mechanisms and processes, e.g., chronic inflammation. Recently, through extensive molecular analyses, it has been pointed out that, contrary to what was previously thought, the microbial community in the vaginal microbiome is definitely more complex and unique to each woman. Moreover, multifactorial influences on its composition are now being emphasized. In addition to hormones, age, host immunity, or genetic predisposition, the primary factors shaping the profile of microorganisms in the vagina also include, for example, diet or lifestyle, which become crucial in the case of PCOS patients for the composition of the microbiome, for example, the gut [51,61]. For women with PCOS, a number of studies have shown the beneficial effects of probiotics on improving metabolic rates and the gut microbiome. Given the potential impact of the gut microbiome on the vaginal microbiome, it is worth considering probiotics, including oral probiotics, in women with PCOS to balance the vaginal microbiome. In PCOS syndrome, dysbiosis of the microbiome of various locations, and thus also the possibility of developing, for example, bacterial vaginosis, is more common than in healthy patients [62].

The importance of oral probiotic therapy on increasing the number of *Lactobacillus* spp. assessed by the Nugent scale in the vaginal microbiome was demonstrated in a study by Petricevic et al. (2008) conducted in a group of postmenopausal women. Initial scores on the Nugent scale ranged from 4 to 6, and after probiotic therapy (with freeze-dried strains of *L. rhamnosus* GR-1 and *L. reuteri* RC-14), 60% of patients showed an improvement of at least two steps on the scale [63].

### 4.6. Lactobacillus spp. Contribution in Vaginal Microbiome

Women with PCOS are indicated to have a lower proportion of *Lactobacillus* spp. in the vaginal microbiome compared to healthy women (Tu et al. 2020). Thus, women with PCOS are potentially more likely to have an imbalanced vaginal microbiome and a higher incidence of BV. In addition, a dysregulated lower genital tract microbiome is associated with fertility and menstrual cycle disorders, which PCOS patients often face (Tu et al., 2020) [7].

*Lactobacillus* spp. in the vaginal microbiome serve as a barrier to infections and have beneficial effects on the innate immune system and maintenance of vaginal homeostasis. Hence, the diversity of the vaginal microbiome should be rather low with the dominance of *Lactobacillus* spp. [51,64]. However, women with PCOS exhibit a highly heterogeneous composition of the vaginal microbiome, with a reduced percentage of *Lactobacillus* spp., and at the same time an increased proportion of potentially pathogenic microorganisms, such as *Gardnerella vaginalis* or *Prevotella* spp. Moreover, in the genital tract of PCOS patients, favorable conditions for such microorganisms are provided by an excessive number of amino acid metabolism pathways, oxidative phosphorylation, and N-glycan biosynthesis [7]. In our analysis, even in PCOS patients without BV found according to routinely used criteria, the vast majority of PCOS patients had *Gardnerella vaginalis* or an elevated pH of the vaginal environment, which in the case of these patients becomes particularly important in terms of more favorable conditions for the development of BV.

In the vaginal environment, the importance of *Lactobacillus* spp. is particularly important due to their action as a barrier to infection due to the glycogen metabolism processes carried out, the production of lactic acid, and the maintenance of a low pH ≤ 4.5. Elimination of *Lactobacillus* spp. results in an increase in vaginal pH and further, among other things, excessive proliferation of anaerobic bacteria and the development of BV, leading, for example, to fertility disorders or premature births [65]. Currently, however, there is growing evidence of heterogeneous effects on the vaginal microbiome within the genus *Lactobacillus*, depending on the characteristics of each species [64]. According to a study by Ravel et al. (2011), five types of community status (CST) are found in women of childbearing age, depending on the proportion and species diversity of *Lactobacillus* spp.: CST I—with *Lactobacillus crispatus* predominating, II—with *L. gasseri*, and V—with *L. jensenii*, all most associated with good health. CST III (with *L. iners*) is ambiguous and can show BV, dysbiosis, or a physiological condition [50]. The clinical picture of BV is mainly characterized by CST IV status, with the predominance of microorganisms such as Gardnerella, Mobiluncus, or Prevotella and the elimination of *Lactobacillus* spp. Sometimes more detailed breakdowns are made for CST IV, including subgroups: A—with *L. iners* and with strict anaerobes; B—with bacterial species related to BV (BVAB); and C—with many various strict and facultative anaerobes [51]. Hence, the mere presence of *Lactobacillus* spp. does not guarantee the stability of the vaginal environment. Therefore, the routinely used assessment of the vaginal microbiome and determination of BV by standard methods should be revised. While microbiological analysis of vaginal secretions with assessment of the presence of *Lactobacillus* spp., or additionally using the Nugent scale, provides some picture of the quantitative presence of *Lactobacillus* spp., it does not allow for its qualitative assessment with consideration of the species of these bacilli. Such tests are also not commonly used in routine diagnostics. Therefore, when analyzing the results of PCOS patients in the present study, it was not possible to determine which *Lactobacillus* species were detected. This fact is important because of the different properties and functions of individual *Lactobacillus* species. Some patients, despite the presence of acceptable amounts of *Lactobacillus* spp., reported symptoms suggestive of BV development. However, this was not reflected in routine diagnostic testing. Some studies (Ravel et al., 2011) indicate cases in which routinely used methods of assessing BV, such as high pH and high Nugent scores, do not provide a clear answer as to the composition of the vaginal microbiome. It turns out that some desirable microorganisms, such as *Lactobacillus* spp., may still be present. An example is *L. iners*, which is probably a species with lower protective capacities in maintaining the stability of the vaginal microbiome [50,66]. In patients with PCOS (Hong et al., 2021), a relationship between testosterone levels and the diversity of the β vaginal microbiome is indicated, in addition to the presence, proportion, and species diversity of *Lactobacillus*, such as *L. crispatus* and *L. iners* [6]. Hence, due to the protective potential for the vaginal environment of the genus *Lactobacillus*, the importance of species identification is emphasized [51]. Such detailed analyses, however, require more sophisticated techniques, such as molecular biology methods, than those used in routine diagnostic microbiological tests to assess the vaginal microbiome of patients, in which precise species identification can be made. However, they are currently rather used in scientific research.

Moreover, as in the case of the group of PCOS patients presented in our analysis, an imbalance of the vaginal microbiome, dysbiosis or already-developed BV may not always produce unambiguous and characteristic symptoms, which generates additional difficulties in assessing and implementing prompt and effective therapy. The individual needs of the vaginal ecosystem depending on a woman’s life stage (premenopausal, perimenopausal, postmenopausal) are also pointed out, resulting in a need for individual selection of probiotic therapies [66]. Due to the growing need to identify species of *Lactobacillus*, detailed diagnostics based on molecular tests may be more and more often included in routine tests, and not only at the level of scientific research. An example of the need for such opportunities is *L. iners* in the vaginal environment. Although they are classified as probiotic microorganisms, attention is drawn to the differences in their activity in maintaining a healthy vaginal microbiome in relation to other dominant *Lactobacillus* species (e.g., *L. crispatus*, *L. gasseri*, *L. jensenii*). The differences concern, for example, the ability to produce lactic acid in the vaginal microbiome. *L. iners*, due to the lack of the gene encoding D-lactate dehydrogenase, produces only L-lactic acid as a result of fermentation of glycogen, and not both D- and L-lactic acid isomers that positively affect the immune system (Witkin et al., 2013; Pramanick et al., 2018) [67,68] and prevent the possibility of developing upper genital tract infections (Beghini et al., 2015) [69]. The ability of *L. iners* to produce only L-lactic acid therefore limits its effectiveness in the fight against pathogens in the vagina [70]. A greater inhibitory potential of the D-isomer of lactic acid for exogenous bacterial infections has been demonstrated than in the case of its L-isomer. [71,72]. In the case of *L. iners*, but also *G. vaginalis*, low levels of or no D-isomer of lactic acid are found in BV, which may translate into an imbalance of both isomers and induction of proteins responsible for the ability of the bacteria to pass through the cervix and initiate infections of the upper reproductive tract [67]. *L. iners* often shows high abundance even in the case of BV [72]. This explains the nature of this microorganism and its limited possibilities of counteracting the development of pathogenic microorganisms such as *Candida* spp. [73]. or *Gardnerella vaginalis*, by lowering the pH of the vaginal environment. *L. iners* occurs less frequently at a low physiological pH of the vagina, when other species of the *Lactobacillus* genus dominate. However, analogically, its presence is found when the pH increases, in the presence of *G. vaginalis*, in pH typical of BV, and it can survive in vaginal dysbiosis conditions [74] when the remaining *Lactobacillus* spp. are substantially absent [75,76]. In addition, the low potential of *L. iners* in preventing the colonization of the vaginal environment by anaerobes is indicated due to its low capacity to produce pyruvate H_2_O_2_ through oxidation [72,77,78,79].

Species of *Lactobacillus* were not identified in the PCOS patients analyzed in our study, as such diagnostics do not include routine vaginal microbiome testing available to the physician. According to routine diagnostics, the presented group of PCOS patients may not have been diagnosed with BV, although clinically it may have been initially suspected; however, the number of *Lactobacillus* spp. was high and therefore did not meet the criterion for a BV diagnosis, although perhaps the presence of *Lactobacillus* spp. alone did not fulfill its beneficial function.

### 4.7. Candida spp. Contribution in Vaginal Microbiome

In addition, *Candida* spp. and *Gardnerella vaginalis* were also found in the presented group of patients independently of the presence of *Lactobacillus* spp., and some studies suggest that the microbiome with a dominance of *L. iners* may be a specific “refuge” for *Candida* spp. in the vagina (for example, [73]). It is also worth noting that as a result of an imbalance of the vaginal microbiome, or dysbiosis, an environment is created that is conducive to the growth of other pathogenic microorganisms and opportunistic fungi, especially Candida (e.g., *C. albicans*, *C. glabrata*, *C. krusei*, *C. parapsilosis*) [51,80]. BV can therefore be accompanied by vaginal candidiasis, generating additional symptoms observed by the patient, which distorts the picture of the original source of the patient’s clinical condition and results only in the empirical implementation of antifungal therapy. Recently, the high value of NAAT-based methods has been pointed out as facilitating both the diagnosis of BV and candidiasis. However, like other molecular tests, they are costly and are currently not widely used in the routine diagnosis of BV [28]. In addition, there is the possibility of BV with associated candidiasis being a result of, for example, antibiotic therapy. A consequence of antibiotic therapy, especially broad-spectrum antibiotics, is the elimination of *Lactobacillus* spp. from the vaginal microbiome, which in turn leads to its imbalance [51]. In the case of our work, in the analyzed results of patients with PCOS, no correlation was observed between the number of patients with *Candida* sp. present in vaginal secretions and an increase in pH or BV (with clue cells). Moreover, in the analyzed PCOS patients, the identified presence of *Candida* spp. in the vaginal microbiome did not lead to automatic elimination of LAB, and in some patients both types of microorganisms were detected.

### 4.8. The Effect of BV on the Fertility of Patients with PCOS

One of the many problems that PCOS patients face is infertility. Often, BV can be a potential, although underestimated, source of infertility. An example confirming the significant role of BV in reduced fertility is the analyses of Salah R.M. et al. (2013) in patients with PCOS and unexplained infertility, in which a higher incidence of infertility and a positive effect of the use of BV therapy on the improvement of pregnancy rates were demonstrated [43].

Moreover, it is suggested that *L. iners* may be even a factor that is unfavorable to pregnancy [72,79], which is a particularly important aspect for women with PCOS, and its detection could be an important part of microbiological diagnosis. Women with PCOS are a special group of patients because of problems directly related to the syndrome, such as hormonal disorders and infertility problems. Hence, the possibility of using *L. iners* as a potential predictive biomarker for BV is being considered, which would enable faster introduction of effective therapeutic options [72].

### 4.9. The Most Commonly Reported Symptoms by PCOS Patients

In the analysis we presented here, one of the most common symptoms that PCOS patients reported seeing their gynecologist with, aside from unpleasant (“fishy”) vaginal odor, was often vaginal dryness. This is a symptom that is also frequently reported by patients struggling with BV and/or developing vaginitis. This condition leads to general discomfort and is an additional factor potentially further affecting the low self-esteem of these patients [8,81,82,83].

## 5. Conclusions

BV appears to be potentially more common in patients with PCOS than in the population of women without the syndrome. This fact can be explained by a predisposition related to its varied course and the presence of several disorders, including hormonal disorders. As a result, women with PCOS often exhibit abnormalities in the vaginal microbiome, with the elimination of *Lactobacillus* spp. and the creation of favorable environmental conditions for the development of pathogens, such as *Gardnerella vaginalis*, affecting reduced fertility or complicating pregnancy.

The detection of *Lactobacillus* sp. as a genus in the routine microbiological diagnosis of the vaginal microbiome, especially in the context of BV diagnosis, without the species identification of *Lactobacillus* spp. (e.g., *L. iners*), does not guarantee protection against its development. This fact should be an important signal for clinicians in the diagnosis of BV, especially in patients with PCOS. It is therefore worth reviewing whether the conventional methods routinely used, including the Amsel criteria and Nugent scoring, based on quantitative assessment of *Lactobacillus* spp., should be enriched with more precise and detailed molecular biology methods.

The evaluation of the vaginal microbiome and its disorders in patients with PCOS deserves special attention. To date, few studies have fully analyzed this issue and elucidated the mechanisms in the vaginal environment of patients with this disorder. Based on our analysis and the results of routine microbiological diagnosis for BV in patients, in our case with PCOS, it seems likely that due to the predisposition associated with the syndrome, BV is more common compared to in healthy women without the syndrome. For women with PCOS, the importance of BV prevalence also increases as a likely unrecognized cause of infertility and/or pregnancy complications. In patients with PCOS, the importance of chronic inflammation, demonstrated in several studies in the gut microbiome, is also highlighted and worth exploring in the context of vaginal microbiome disorders. Given the entire spectrum of abnormal symptoms and serious consequences, both hormonal and metabolic (e.g., diabetes, insulin resistance), as well as reproductive, emotional, and psychological, such as depression, a closer examination of the vaginal microbiome and its abnormalities, and the genesis of the potentially more frequent development of BV, may shed a different light on PCOS patients and their therapies.

There appears to be a potential link between PCOS syndrome and the development of BV, but it requires further research that could help clinicians make faster diagnosis and effective treatment.

### Limitation of the Study

Analysis of the results of studies of patients with PCOS suggests a higher prevalence of abnormalities in the vaginal microbiome, influencing the potentially more frequent development of BV (bacterial vaginosis) and, consequently, potentially the fertility of patients with the syndrome. However, the direct mechanisms and causal relationship remain unknown and require further study. A limitation of the present study is the methods used in routine microbiological diagnosis. These include quantitative methods for *Lactobacillus* spp. which, however, may not give clinicians a complete picture of the changes occurring within the vaginal microbiome of a patient with PCOS. Therefore, it may be worth considering extending diagnostics to include more precise testing via molecular methods.

## Figures and Tables

**Table 1:** Reported PCOS patients’ symptoms.

The Most Commonly Reported Patient’s Symptoms	Reference
vaginal burning and/or dryness	[15,16,17]
swelling, redness, itching of the vulva	[15,16,17]
“abnormal”/watery/grey appearance of vaginal discharge	[15,16,18]
unpleasant/“fishy smell” of the discharge	[15,17,18]
inaccurate treatment of recurrent infections	[19]
discomfort or dyspareunia during sexual intercourse	[20,21]

**Table 2:** BV criteria according to Amsel and Nugent scoring systems.

Criteria of BV:
**Amsel** [22]:
thin, white-grayish, milky homogenous vaginal discharge
pH > 4.5 of the vagina
amine odor (“fishy”) in whiff test
“clue cells” (epithelial cells covered with bacteria)
**Nugent** [9]:
low Nugent score (0–3), healthy vaginal microbiota
medium Nugent score (4–6)
a high Nugent score ≥ 7, positive diagnosis for BV

**Table 3:** Criteria applied to the selection of the patients.

Inclusion Criteria for Analysis:	Exclusion Criteria for Analysis:
- reproductive age (23–39 years old)	- thyroid diseases - Cushing’s disease - diabetes - hyperprolactinemia - congenital adrenal hyperplasia
- Established PCOS according to the Rotterdam Consensus criteria; found at least two of the following symptoms: (1) oligo- or amenorrhea; (2) clinical or chemical hyperandrogenism; (3) and/or polycystic ovaries as determined by transvaginal ultrasonography [14]	
- pH of vaginal secretions pH ≥ 4.4	- pH of vaginal secretions between 3.8 and 4.2
- presence of Amsel criteria guidelines	- without any BV “markers”
- Nugent score medium (4–6) and high (≥7)	- low Nugent score (0–3)
- patients reporting a history of symptoms, e.g., redness, itching of the vulva, “abnormal”/watery/gray appearance of vaginal discharge, unpleasant/“fishy” odor of discharge, burning and/or dryness of the vagina, swelling	- no symptoms, routine gynecological check-up, e.g., before a planned pregnancy

**Table 4:** Contingency table of observed values of patients with clue cells and *Gardnerella* present.

pH	Clue Cells	G+ Multiple	Total
4.4	8	5	13
4.6	34	27	61
4.8	81	115	196
5.4	68	69	137
Total	191	216	407

**Table 5:** Logistic regression model (LRM) matrix.

Sample_number	Ph_group	Presence/Absence
Sample_1	4.4	1
Sample_2	4.4	0

**Table 6:** Contingency table of observed values of patients with absence of lactic acid bacteria and increased levels of leukocytes at different pH levels.

pH	LAB None	Leu > 10	Total
4.4	12	2	14
4.6	18	10	28
4.8	94	57	151
5.4	84	53	137
Total	208	122	330

**Table 7:** Relationship between the detection of the *Candida* species and some of the tools used in diagnosing vaginal discomfort.

pH	Clue Cells	Leu > 10	LAB None
4.4	8	2	12
4.6	34	10	18
4.8	81	57	94
5.4	68	53	84
Total	191	122	208

## Data Availability

Data are contained within the article.
